# A Systematised Review of the Health Impact of Urban Informal Settlements and Implications for Upgrading Interventions in South Africa, a Rapidly Urbanising Middle-Income Country

**DOI:** 10.3390/ijerph16193608

**Published:** 2019-09-26

**Authors:** Amy Weimann, Tolu Oni

**Affiliations:** 1 Research Initiative for Cities Health and Equity, Division of Public Health Medicine, School of Public Health and Family Medicine, University of Cape Town, Cape Town 7925, South Africa; 2 African Centre for Cities, University of Cape Town, Cape Town 7701, South Africa; 3 Medical Research Council Epidemiology Unit, University of Cambridge, Cambridge CB2 0QQ, UK; 4 Stellenbosch Institute for Advanced Study, Stellenbosch 7600, South Africa

**Keywords:** informal settlements, health, South Africa, upgrading, social determinants of health, literature review

## Abstract

Informal settlements are becoming more entrenched within African cities as the urban population continues to grow. Characterised by poor housing conditions and inadequate services, informal settlements are associated with an increased risk of disease and ill-health. However, little is known about how informal settlement upgrading impacts health over time. A systematised literature review was conducted to explore existing evidence and knowledge gaps on the association between informal settlement characteristics and health and the impact of informal settlement upgrading on health, within South Africa, an upper-middle income African country. Using two databases, Web of Science and PubMed, we identified 46 relevant peer-reviewed articles published since 1998. Findings highlight a growing body of research investigating the ways in which complete physical, mental and social health are influenced by the physical housing structure, the psychosocial home environment and the features of the neighbourhood and community in the context of informal settlements. However, there is a paucity of longitudinal research investigating the temporal impact of informal settlement upgrading or housing improvements on health outcomes of these urban residents. Informal settlements pose health risks particularly to vulnerable populations such as children, the elderly, and people with suppressed immune systems, and are likely to aggravate gender-related inequalities. Due to the complex interaction between health and factors of the built environment, there is a need for further research utilising a systems approach to generate evidence that investigates the interlinked factors that longitudinally influence health in the context of informal settlement upgrading in rapidly growing cities worldwide.

## 1. Introduction

In Africa, and across the global South, urbanisation and urban growth are dramatically restructuring the nature of cities. The majority (55%) of Sub-Saharan African urban dwellers now live in slums and informal settlements—a proportion that is notably larger than the global average (30%) and other developing regions including South Asia (31%) [1]. Informal settlements are characterised by poor housing that does not comply with building or planning regulations, a lack of sufficient basic services, inadequate healthcare and other public amenities, and housing that offers no tenure security for inhabitants [2]. The term is often used interchangeably with ‘slums’, however slums are characterised by dilapidated housing with inhabitants that suffer from deprivation in one or more of the following categories: access to potable water; security of tenure; access to sanitation; and an adequate living environment [2].

These environments pose an unequal threat to the health of the urban poor and contribute to the spread of infections due to poor sanitation; a higher incidence of respiratory infections and conditions, including asthma; injuries; and the prevalence of mental disorders, including depression and stress [3,4,5,6], thereby increasing the burden of acute and chronic infectious and non-communicable conditions (NCDs) [3,7,8]. Moreover, inhabitants of informal settlements experience social and spatial marginalisation and thus are confronted with an increased risk to mental and overall wellbeing [2]. The need to address these deficits is underscored in the Sustainable Development Goals (SDGs)—a set of global aspirations for attaining sustainable development by 2030—and specifically through the global call to provide adequate dwellings and liveable environments in SDG 11 which seeks to ‘Make cities inclusive, safe, resilient and sustainable’ by ensuring, inter alia, access to adequate, safe and affordable housing for all [9]. The UN-Habitat III New Urban Agenda [10] contributes to the achievement of SDG 11 by committing to ensuring, among other things, the realisation of the right to adequate housing and safe living environments by supporting local and national level policies.

International development agendas and reports such as the New Urban Agenda [10], the Commission on the Social Determinants of Health’s Closing the Gap in a Generation: Health Equity through Action on the Social Determinants of Health [11], and the World Health Organization (WHO) Housing and Health Guidelines [12] highlight the influence that living environments play in shaping and determining health and wellbeing. While, SDG 3 seeks to ‘Ensure healthy lives and promote wellbeing for all at all ages’, the interaction between SDG 3 and SDG 11 needs to be acknowledged, as inadequate living environments (SDG 11) will need to be addressed if health and wellbeing is to be achieved for all (SDG 3) [9]. While there is an international emphasis on improving poor living environments in order to address health, it is unclear how subnational and local built environment interventions specifically impact on health outcomes over time.

### 1.1. The Conceptual Link between Informality and Health

The urban system is comprised of a complex network or assemblage of interconnecting factors, both human and non-human, that act on and react with each other in response to changes and feedback loops [13,14]. One component of the urban system is housing. The notion of urban assemblage is relevant to the concept of housing when a dwelling is considered as being more than just a built structure; it is also inhabited, and a place of urban belonging, as well as a structure that could have been built in line with political or civil society priorities, or in response to human need [14]. Therefore, housing is a product of an assemblage of urban human and non-human interconnections, and therefore should not only be considered as a physical, structural concept.

Housing, and its complexities, is one of the many underlying factors within an urban system that are able to shape and determine human health, defined as complete physical, mental and social wellbeing [15]. This is the underpinning notion of the socio-ecological model of health, which highlights that individual health and wellbeing are greatly influenced and determined by a complex interaction of underlying factors [16,17]. These factors, also referred to as social determinants of health, include those related to the quality of the surrounding living environment; stressors encountered through work; as well as behavioural and lifestyle choices that influence health in addition to underlying genetic influences [11]. Combining the concepts of health and housing—examples of components within an urban system that interact with each other—the WHO suggests a holistic approach to exploring the housing component. Beyond just the house structure, it considers four overlapping and interrelated dimensions of housing that contribute to health and wellbeing: the physical housing structure, the psychosocial and cultural home environment, the physical characteristics of the neighbourhood environment, and the social environment and services within the community [18,19]. Within the context of informal settlements, examples of likely characteristics within each domain include dilapidated or inadequate shelters with inadequate ventilation and high levels of indoor pollution (house); high socio-economic stress experienced by families (home); high-risk areas for natural disasters due to poor location (neighbourhood); and social stresses such as high crime or gender discrimination (community) [2,20].

### 1.2. Informality and Health Globally and in South Africa

It has been argued that urban studies theory has largely been based on urbanisation trends of the Global North, thereby highlighting the need for continued research into the prominent features of Global South cities, such as proliferating of informal settlements, and how these may, in turn, be contributing to the complex urban challenges in developing countries [21,22]. Corburn and Sverdlik [23] conducted an evaluation of informal settlement (or “slum”) upgrading on health in the context of Asian, African and South American cities using selected peer-reviewed studies and grey literature. Of the 19 studies selected, eight were specific to the context of Africa. Four of these investigated informal settlement upgrading in Kenya, two in Tanzania and two in South Africa (SA). Only the studies conducted in Tanzania discussed health variables, albeit very broadly [23]. Neither of the two studies in SA explicitly sought to measure the impact of upgrading on health profiles, instead focusing on differences in housing and neighbourhood characteristics between formal upgrades and shacks, and on boundary types and disputes within settlements. These results highlight a lack of health impact evaluations of informal settlement upgrading in the Global South.

SA is one of the most urbanised countries in sub-Saharan Africa with 66% of the population residing in urban areas and is continuing to experience an increase in the number of urban inhabitants [24]. Like many other countries in Africa and the global South, this urbanisation process is often unplanned, and characterised by significant spatial inequalities, with high levels of health disparities [25]. This makes SA an important case study for cities in the Global South that are rapidly urbanising. Many of the consequences accompanying urbanisation, such as the large numbers of informal settlements and the strain on urban resources and increased growth in the housing backlog, are visible throughout South African cities, suburbs and peri-urban areas. The development of informal settlements in SA can also be attributed to a history of racial discrimination and segregation through the legacy of apartheid, which fuelled inequality and confined the majority of the population of colour to poor, underserviced living environments through the Group Areas Act (No. 41 of 1950) [26]. Furthermore, inefficient strategies for urban management, corruption and ineffective policies have also contributed to the development and growth of informal settlements, among other reasons [27]. In South Africa, “informal settlements” are generally defined as areas containing unregulated and unplanned dwellings where inhabitants lack secure tenure and thus generally lack access to adequate basic services prior to government intervention [28].

It has been argued that the eradication of slums should happen indirectly, as a result of prioritising the improvement of informal settlement environments and conditions through in situ upgrading, which aims to reduce the amount of disruption to people’s lives [29,30]. However, little is known about the impact of informal settlement upgrading in the Global South on population health trends over time. Aligned with the SA National Upgrading of Informal Settlements Programme, in the National Housing Code, the SA informal settlement upgrading process can be divided into four stages [31]. Through various assessments, sub-national government may determine which phase of development the informal settlement is able to reach. Phase 1 includes project registration in which suitability assessments are conducted and business plans are developed [31]. Phase 2 seeks to initiate the project, for example through the acquisition of land, if needed; registering households within the site, and providing interim basic services, such as water and sanitation [31]. Stage 3 and 4 are formalisation stages in which permanent engineering solutions are provided (stage 3), such as a subdivided plot with individual services, and formal top-structures are developed (stage 4), provided that the beneficiary’s application for a government housing subsidy has been successful [31]. This upgrading process primarily deals with improving the structure and services of the house and neighbourhood—two of the four interrelated dimensions of housing that the WHO has identified for a holistic approach to health [19]. The remaining dimensions of the home environment (psychosocial, cultural and socioeconomic factors) and the community (wider social setting) are often not considered.

Given the geographical and conceptual knowledge gap on the health impact of informal settlement upgrading, this review, using the case study of South Africa, focuses on exploring available literature and research to better understand the state of health in South African informal settlements using the WHO’s four dimensions of housing [19] and to investigate potential influences of informal settlement upgrading on the health and wellbeing on vulnerable groups of residents. In this study, health is defined according to the WHO’s [15] holistic definition, which recognises health as a state of physical, social and mental wellbeing.

## 2. Materials and Methods

**Figure 1:**
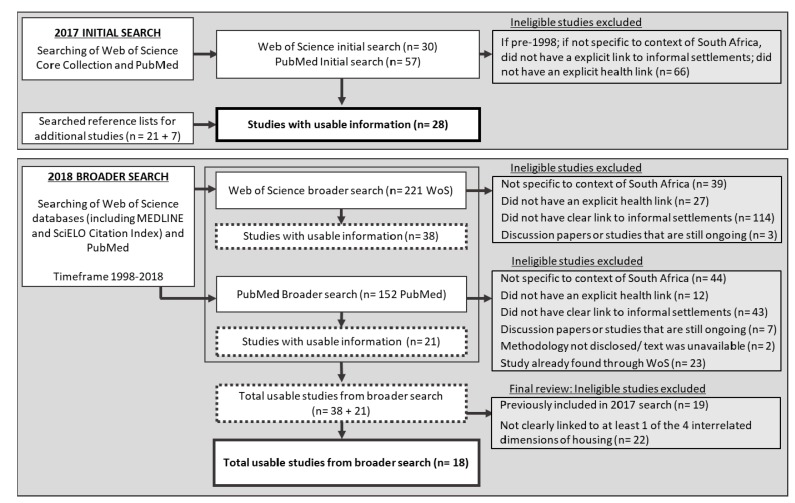
Flow Diagram of search steps.

Between March and June 2017, a systematised review of peer-reviewed studies was conducted. A systematised review includes some components of a systematic review, as it seeks to systematically review available peer-reviewed literature on the topic of interest, however it is not exhaustive, is useful when there is no intervention being evaluated and if there is an absence of multiple reviewers [32]. Peer-reviewed research studies seeking to measure the association between informal settlements and health in SA and evidence on the health impact of informal settlement upgrading, were investigated (Figure 1). As the aim of the literature review is not to provide an evaluation of a specific intervention, as is the case with systematic literature reviews, the quality and validity of each study was not required to be critiqued beyond an initial judgement.

Using two databases, Web of Science and PubMed, an initial search was conducted using the terms “UPGRADING” AND “INFORMAL” AND “HEALTH” AND “SOUTH AFRICA”. The abstracts of the articles were reviewed, and studies were included if they met all of the following criteria: the study was specific to the context of SA; the study had an explicit link to informal settlements and could be linked to one or the four dimensions of housing (i.e., the house, the home, the neighbourhood infrastructure or the community); and if the study had an explicit link to health within the context of informal settlements. Studies were excluded if they were discussion papers, ongoing studies or if their methodology was not well described; if the study was only vaguely linked to general poverty or homelessness (not explicitly linked to informal settlements); and if the full text was not available. Studies were excluded if they were published earlier than 1998, as the South African National Housing Code, which incorporates an informal settlement upgrading plan, was implemented in 1997. A search strategy has been provided detailing the search terms used, the inclusion and exclusion criteria and the included references (available as a supplement).

The Web of Science database produced three results but only two studies were included in this study, as the third study was a discussion paper on international housing legislation. One of these was a mixed-methods review of research for the case-study site of Freedom Square in Bloemfontein, SA [33] and the other was a cross-sectional study [18] which sought to investigate self-reported health changes in response to the upgrading of Imizamo Yethu informal settlement in Cape Town. The search results using the PubMed database produced no results and thus the search terms were broadened to “UPGRADING” AND “INFORMAL” AND “HEALTH”. This returned 23 studies from Web of Science and eight studies from PubMed, in addition to a literature review conducted by Corburn and Sverdlik [23] which was not included in the final set of articles. However, only three of these studies from Web of Science [18,33,34] and none from PubMed were found to be specific to the context of SA informal settlements. Of the three identified from Web of Science, none of these were longitudinal studies. By providing evidence of temporality, this study design would enable causal inference on the impact of informal settlement upgrading on health, confirming the evident paucity of studies that seek to measure the impact of informal settlement upgrading on health within the context of SA. Additional combinations of search terms, which also included “SLUMS” and “WELLBEING” and their plurality and variations in spelling, were tested, however no additional studies were found. Therefore, the scope of the search was modified and broadened.

Due to the paucity of studies measuring the health impact of informal settlement upgrading in SA, the scope was broadened to review any peer-reviewed studies in SA that investigated the state of health or health risk factors in informal settlements (able to be linked to the WHO’s four dimensions of housing [19]), or across housing typologies, which must include informal settlements. Studies were also included if findings suggested possible links between informal settlement characteristics and health variables. Applying the same inclusion and exclusion criteria used in the initial search and reviewing the abstract and full-text, studies from the search results were considered for inclusion on a case-by-case basis, specifically if the research was based on a single case study and results difficult to generalise, or if the research only investigated a link with poverty. Using the Web of Science Core Collection and PubMed databases, variations of the search terms INFORMAL SETTLEMENT(S), INFORMAL, SOUTH AFRICA, HEALTH, SLUM(S) and WELLBEING were used to create additional searches. From the total 87 studies that were found, only 21 were identified as relevant and used to inform this review (Figure 1). In addition, the reference lists within these 21 studies were searched in order to identify any other studies of relevance. In total, 28 studies were identified (Figure 1), which were in the form of experiments, retrospective case note reviews, case studies, cross-sectional studies, randomised control trials, qualitative studies, and systematic reviews.

Between June and October 2018, an additional search was conducted to identify any recent studies or studies that were previously missed due to the selected search terms and databases. The search was expanded to all databases linked with Web of Science, including MEDLINE and SciELO Citation Index. Additional combinations of search terms were used, as detailed in the search strategy in the Supplementary Material (Tables S1 and S2). An additional 18 studies were found that could provide further insight into the state of health in South African informal settlements (Figure 1). Therefore, a total of 46 studies were used as the foundation of the review of the state of health in South African informal settlements.

## 3. Results

Although there is a growing interest in research investigating health in the context of informal settlements, our search results identified a paucity of literature investigating health impacts of informal settlement upgrading. Only one longitudinal study was identified that specifically monitored changes in health in response to improvements made to the informal settlement or township, through the investigation of the level of satisfaction with quality of life with informal settlement improvements over time [35]. As part of a wider longitudinal cohort study, two studies measured noise pollution effects in the Western Cape Province of SA [36,37]. However, neither of these studies explicitly investigated an association between the informal settlement living environment and health. Nevertheless, they were included in the review as they provide insight into an important risk factor for informal settlements. We identified studies that investigated associations between informal settlement characteristics and health, which provides insight into possible health challenges of informal settlements prior to upgrading interventions. The associations will be discussed using the four dimensions of housing.

One qualitative research study investigated residents’ perceptions of safety within their community after upgrading had occurred, however this was not a longitudinal study and health variables were not explored [38]. Most studies mentioned a possible association between health outcomes and elements of the informal settlement environment, but very seldom investigated these, as it was argued that causality is difficult to prove in this context [39]. Moreover, of these 46 identified studies, only four discussed NCDs, excluding mental health which varied in definition and measurement between studies [40,41,42,43]. An additional four of the selected 46 studies sought to explore aspects relating to mental wellbeing [18,44,45,46].

An overview of the characteristics and possible associated health conditions for the informal settlement living environment is displayed in Table 1. The findings of this review will be discussed within the WHO’s [19] four interrelated housing dimensions, as used by Shortt and Hammett [18]. As these dimensions overlap due to the complexity of the informal settlement living environment, accordingly, the findings were also found to be relevant to multiple dimensions.

### 3.1. The Physical Housing Structure

The house structure has been linked with certain health outcomes. Mathee et al. [50] investigated health inequality in Johannesburg, South Africa across five housing typologies; informal settlements, traditional and modern low-cost housing settlements, mixed-use housing and high-rise city housing. Housing typologies were associated with differences in health, social and psychosocial stresses, and concerns about noise, violence and perceptions of safety were most often reported by the informal settlement residents. Compared to the other housing typologies, informal settlement households most often reported concerns about damp conditions, were least likely to use electricity for cooking, and most likely to have had a recent acute infectious illness (vomiting or diarrhoea) [50].

Crowding within houses is a common feature in informal settlements and is suggested to increase the risk of exposure to, and transmission of infectious diseases, such as tuberculosis (TB), and increase the risk of mental illness (Table 1) [45,48,51]. A qualitative investigation into the mental health of orphans and vulnerable children in a municipality in the Free State province in SA found a significant (*p* < 0.05) association between mental health difficulties and crowded housing conditions [45]. Despite this association being weak (OR = 1.09: CI 1.00–1.19), it is supported by the findings of many other global studies investigating housing and mental health of children [68]. The association between informal housing and mental illness was confirmed by Shortt and Hammett [18] who investigated the impact of housing typologies on the wellbeing of residents and confirmed a strong association, even after controlling for possible confounding variables (OR = 5.739, CI = 1.284, 25.641). Moreover, while the multivariate analysis did not confirm any other associations, bivariate analysis indicated that injuries were three times more likely to occur in shacks, possibly due to crowding and a lack of designated cooking space [18].

In a study investigating the cause of burns in a cohort of children admitted to a Cape Town hospital, findings show that most burns to children occur in informal settlements [49]. The cost and lack of access to electricity and the subsequent use of other sources of energy for cooking, heating and lighting, such as paraffin, coal and wood, as found by other studies [57,61,69], is likely to be a contributing factor to injury and burns risk [58]. Moreover, the use of chromated copper arsenate treated firewood for household purposes is likely to expose informal settlement residents to carcinogens [56]. Informal dwellings are often poorly designed and small in size, increasing the risk of burns and smoke inhalation [49].

One study, conducted in the poorest areas of Johannesburg, found the counterintuitive finding that respondents in informal dwellings were healthier and had significantly fewer chronic health conditions (a composite measure comprising high blood pressure, diabetes, TB, asthma, heart condition or stroke) than respondents living in formal dwellings [40]. The study suggests that this finding is likely to reflect a multifaceted relationship between housing and chronic health conditions, and further suggests a possible “healthy migrant” phenomenon due to the informal housing sample comprising a large number of migrants [40]. The “healthy migrant” phenomenon suggests that migrants are selectively healthier and are thus able to better tolerate poor living conditions [40]. However, the self-reported nature of the health data could account for the lower reports of disease and illness. Nevertheless, this finding identifies a need for a systems approach to understanding the contextual interactions between various housing components and health.

Findings from a review of two decades of literature and research investigating the association between health and urban development in SA, suggested that the upgrading of housing typology from shacks to subsidised housing may lead to improvements in noise, violent crime, safety, as well as reductions in alcohol and substance abuse [70]. It has been suggested that improvements to noise alone can have health benefits, such as improved sleep and mental health, thus increasing productivity at school and work [36,37,48]. However, with the ever-increasing housing backlog in SA, the hopes of obtaining quality low-cost housing for many informal settlement households are diminishing and the living conditions of low-cost housing are not always an improvement. When comparing low-cost housing conditions with backyard informal dwellings in Imizamo Yethu in Cape Town, a study [18] found that damp and mould were equally present in both typologies. Mould is particularly concerning as global studies have found associations between mould and respiratory infections, allergies and asthma [71]. In addition, many residents are unable to afford maintenance and cleaning costs for their dwelling units, resulting in mould growth, and the presence of pests [48]. Pests, such as rats, ticks, bedbugs, cockroaches, mosquitoes, and fleas are common to informal settlements [52,53,54,55]. In a cross-sectional study investigating household pesticide use, 89% of children exposed to pesticides and presenting with atopic dermatitis were from informal settlements [52].

Despite possible health risks associated with improved housing, the only identified longitudinal study of residents in an informal settlement upgrading site in Soweto, Johannesburg showed a significant association (*p* < 0.05) between housing status, particularly security of tenure, and personal quality of life over time [35]. The study investigated how informal settlement residents rated their personal and environmental quality of life from 1999 to 2002, during the implementation of an upgrading housing project. This upgrading process entailed households being relocated from an informal settlement into an improved housing estate. Four household groups were identified, namely households that were relocated, households waiting to be relocated, households having obtained tenure, and households still living in the informal settlement [35]. Comparisons were made between these groups for the years of 1999 (prior to intervention), 2001 and 2002. The overall findings revealed that residents with an allocated site tenure experienced higher levels of satisfaction compared to informal settlements residents [35]. In addition, the level of one’s satisfaction with their physical house influenced their level of satisfaction with the surrounding neighbourhood [35]. The level of satisfaction with life was also found to be associated with perceived health. Despite these interesting findings, a four-year timespan might not be long enough to truly capture changes in health or quality of life.

### 3.2. Neighbourhood Characteristics and Services

The findings from a literature review investigating health and urbanisation over 20 years in SA, highlight the importance of water services and sanitation for addressing infectious diseases and improving wellbeing [33]. One study argued that a lack of water onsite in informal settlements and the subsequent collection and storage of water in containers increases the risk of infectious diseases [72]. This was supported by Goebel et al. [60] who identified a positive association between bloody diarrhoea and storing water in containers (*p* = 0.059). The significance of the association increased if the containers were not thoroughly cleaned (*p* < 0.0001) [60]. However, a critique of this study is that the statistical methods used for determining the associations between health and housing characteristics were not disclosed and odd ratios were not used or reported. A lack of infrastructure in a Johannesburg informal settlement study site resulted in approximately a quarter of households reportedly having to use the bushes as their toilet [61]. Not only does this create an unhygienic environment, particularly if these areas are located near streams or are prone to water runoff, but it also increases vulnerability to crime and gendered violence, particularly at night [61]. Unclean, polluted environments, which are attributed to a lack of sanitation and inadequate services, are suggested to place added pressure on the immune system of TB and HIV-infected people [72]. In one Cape Town informal settlement known to have high HIV prevalence, the high level of TB risk due to overcrowding and limited access to healthcare has likely exacerbated the HIV and TB co-epidemic [47].

Another concern with the neighbourhood characteristics of informal settlements relates to exposure to contaminated water, either through an informal settlements’ proximity to rivers or through the accumulation of water in the area due to a lack of drainage. One study sampled water from surface runoff in four Cape Town urban poor communities comprising low-cost housing and informal backyard dwellings and found high levels of faecal bacteria and *Escherichia coli* (*E. coli*) [62]. It was suggested that this might explain the high levels of diarrhoea cases that were reported by households in the study. Adjacent rivers can expose residents of informal settlements to a high level of *E. coli*, other pathogens, and chemicals due to the surface runoff from the settlements, leading to serious health consequences, including bacillary dysentery and cholera in some instances, and even cancer in areas with high levels of Uranium and Arsenic in rivers [42,43,63,73,74,75,76].

It has been argued that ineffective services and a lack of sanitation in informal settlements is negating the possible health benefits that other informal settlement improvement strategies are supposed to have [77]. Marais and Ntema [33] stress the importance of maintaining infrastructure in informal settlements, as deterioration in quality will have negative consequences for health. While wider national or sub-national interventions to address health outcomes often yield favourable results, local level initiatives and interventions can supplement wider health interventions. Box 1 describes an example of a systems-approach that can be applied to the other domains of housing and other health challenges. The panel describes the example of addressing diarrhoeal disease deaths in Cape Town, Western Cape, SA by using local interventions to supplement national efforts.

Box 1 **Local interventions to address diarrhoeal deaths in informal settlements in Cape Town.**

The warm Western Cape summer is accompanied by a seasonal rise in reported diarrhoea cases, with 25,000–30,000 cases for children under 5 years annually [78]. However, there has been a 58% decline in diarrhoeal deaths for children under 5 years between 2009 and 2013 [79]. Although national interventions have been essential to reducing diarrhoeal deaths, such as the rotavirus vaccine roll-out in 2009, and the encouragement of breastfeeding, other local level interventions have supported and contributed to these efforts [80].

One such intervention is the use of geographic information systems to identify diarrhoea death ‘hot spots’ in informal settlements and the targeting of these hot spot areas for health and hygiene education [79,81]. In addition, home visits are conducted by local government (LG) environmental health officers in Cape Town for each diarrhoeal death to evaluate the state of the living environment and to escalate service delivery issues to the LG [81]. Furthermore, LG environmental health officers make weekly visits into informal settlements to identify and report any water and sanitation maintenance issues, and monthly meetings are held for each Cape Town sub-district to discuss approaches to address key informal settlement concerns [81]. These local level interventions, together with the wider roll-out of the rotavirus vaccine and breastfeeding campaigns, have contributed to a noticeable reduction in under-5 diarrhoea fatalities [81]. This provides an example of effective intersectoral local government strategies, taking a systems-approach to address diarrhoea deaths in children living in informal settlements.

### 3.3. Psychosocial and Cultural Home Environment

Poverty and factors relating to low socioeconomic status are likely to increase levels of stress and feelings of depression [67]. While the causality of the relationship between housing and depression or stress is not yet understood [45], it is likely to impact on how the residents perceive their ‘home’. In a cross-sectional study, only a third (32.8%) of households in a Johannesburg informal settlement were satisfied with their place of residence [61]. A study investigating the prevalence of symptomatic depression in young people living in two South African informal settlements found that 57.9% of young females and 49.5% of young males (18–30 years) were depressed [44]. Depression and other mental health problems in informal settlements was also shown to be related to general socioeconomic factors such as food security and employment [44,46].

However, a cross-sectional study investigating the mental health of orphans and vulnerable children in a municipality in the Free State, unexpectedly found that the likelihood of having mental health issues decreased when residents had access to an outside tap and toilet and lived in an informal dwelling [45]. Marais et al. [45] suggested that improved mental health could be linked to poor households moving into urban informal settlements from crowded areas outside the city that were historically designated for certain racial groups during the Apartheid era, in order to be closer to urban employment opportunities. Another factor is that moving to informal settlements from rural areas may provide a sense of hope for obtaining government subsidised housing. However, it is also likely that other factors were not taken into account that might explain these findings, such as recent environmental or service delivery improvements within the selected informal settlements or educational interventions.

### 3.4. Social Environment

Very few studies were found that address the social environment of informal settlements. However, one study investigated health impacts for female-headed households (FHH) in contrast to other headed households (OHH) in informal settlements [60]. The results showed that, while 57% of deaths in FHH are attributed to illness, FHH were more likely to accommodate old relatives and also experienced higher levels of death due to old age (14%) and accidents (7%), when compared with OHH [60]. In the OHH, the vast majority of deaths were attributed to illness (85%) and no deaths were caused by accidents or old age [60]. Furthermore, the study found that working adults and school-going children were often tasked with looking after ill household members, more so in FHH than in OHH, and subsequently were absent from school and work [60]. This study highlights gender-related inequalities that may influence health patterns, and the importance of applying a gender lens to informal settlement improvement strategies.

Informal settlements are suggested to be lacking in social relationships and networks [47]. These perceptions of a lack of community may be further aggravated when certain households are selected to receive government subsidised housing [18]. The study revealed that informal housing residents who did not receive a form of low-cost housing from the government felt excluded and experienced increased tension and a subsequent deterioration in community cohesion. Moreover, one study found that poor housing increases levels of frustration, which is a possible cause of crime and violence, alongside alcohol use and unemployment [59]. In particular, data from a cluster randomised control trial conducted across informal settlements in Durban, SA [82] suggest that children living in informal settlements experience a high vulnerability for violence and trauma.

In a qualitative study exploring residents’ perceptions of safety in informal settlements that have recently undergone upgrading, most respondents who received a formal house reported they still felt unsafe in the community, where there was a high level of crime and gang violence [38]. Of note, residents who did not receive formal housing but instead experienced informal settlement reblocking felt that the community was safer and that there was a greater sense of community cohesion [38].

Another social aspect emerging in the literature was intimate partner violence (IPV) [83,84]. A study based on the premise that poverty, inequality (including gender inequality) and weak social networks in urban informal settlements are linked to HIV-risk and IPV behaviour, used a cluster randomized control trial to evaluate interventions for reducing HIV-risk behaviour and IPV in informal settlements [64,65,85]. The findings suggest that young men living in informal settlements, who are financially insecure, would use violence as a way to re-establish their masculinity and gain respect, as their economic status left them perceived as being dependents or feeling like children [65]. Therefore, IPV is prevalent in these settings and has particular health implications for women, as they are less likely to negotiate safe sex, have a higher risk of contracting HIV and less likely to adhere to anti-retroviral medication [64]. However, one study examined cross-sectional data for adults between 18 and 23 years living in informal settlements and found that material deprivation, defined as deprivations in food, housing and healthcare, increases the risk of high-risk sexual behaviour for both men (adjusted OR = 1.20; 95% CI = 1.10, 5.58, 3.28) and women (adjusted OR = 1.43; 95% CI = 1.35, 3.28) [66].

## 4. Discussion

While research in the context of SA broadly recognises the influence that urban and intra-urban environments have on health outcomes, there is a lack of evidence on the impact that improvements and upgrades to informal settlements have on residents’ health and wellbeing over time. This is concerning, as it likely reflects the administrative siloes within which the housing and health sectors operate [86,87], despite the fact that upgrading of informal settlements through improved housing and services can and should indirectly improve health and wellbeing. The need to improve health through sustainable housing development is emphasised in the global policy landscape. For example, the International Council for Science suggests that access to adequate housing is likely to support international efforts to reduce child mortality, exposure to infectious diseases, NCD prevalence, and improve the quality of the living environment [88]. As SA is signatory to these global policies, it is noteworthy that SA is not monitoring the health impact of informal settlement upgrading.

### 4.1. Implications for Upgrading Informal Settlements

The reported findings from this systematised literature review suggest direct and indirect associations between certain physical conditions in informal settlements and health outcomes. Direct associations include increased exposure to pathogens due to crowding and inadequate sanitation and polluted environments; examples of indirect associations include the influence that the physical environment has on mental wellbeing, community and social dynamics, and socioeconomic factors that mitigate or increase health risks. Health and housing have been shown to be intrinsically linked and the literature has further suggested a strong association between people’s satisfaction with life and conditions of health and housing.

A question rising in the field of medical geography and urban health, is whether improvements to housing truly result in improvements to health and wellbeing [39,70]. While there is a vast body of literature associating improvements to housing with health improvements, there are few studies that identify only a small association between housing development and health [70]. Therefore, as the findings in this paper have highlighted the associations between informal settlement characteristics and health outcomes, the following recommendations are based on the notion that improvements to informal settlement characteristics are likely to improve health.

#### 4.1.1. Crowding

Crowding is shown to be associated with mental illness, particularly in children and an increased risk of exposure to infectious diseases such as HIV and TB. Moreover, fires spread easier in high density areas which increases the risk of burns and injury. Informal settlement upgrading should seek to minimise crowding both in housing units and in settlements.

#### 4.1.2. Ventilation

As mould and moisture within the home have been associated with an increased risk to asthma, allergies and respiratory illnesses, improvements to ventilation should be a priority for informal settlement upgrading. Improvements could include additional windows for dwellings, reorganising the informal settlement to improve ventilation between structures, or health awareness campaigns related to mould and damp within the home.

#### 4.1.3. Services and Sanitation

The findings highlight the importance of basic services, such as toilets, access to water and electricity, for health outcomes. However, a lack of sanitation in informal settlements may negate any opportunities for health improvements, even if all other forms of basic services are provided. Moreover, unless suitable drainage for wastewater is provided and household refuse is collected regularly, the environment will continue to expose residents to sources of infection and disease, and particularly increase the pressure placed on the immune systems of ill residents. As many residents of informal settlements live in a state of poverty, the costs of accessing primary healthcare and adhering to medication places an added burden on their socioeconomic status. Moreover, improved access to primary healthcare, through an increase in the number of clinics and an increase in affordable transport are important for improving health.

#### 4.1.4. Consideration of Vulnerable Groups

Of the studies available, there was very little focus on identifying vulnerable groups within the informal settlement environment. Overall three vulnerable groups were identified in the literature, namely females, HIV/TB-infected people, and children. Females are vulnerable to both socioeconomic and health impacts in the context of IPV and FHH. Gender-related inequalities in health, particularly in the context of IPV and FHH need to be considered when upgrading informal areas. The weaker immune systems of people with HIV and TB, as well as other chronic infectious and non-communicable diseases, increase vulnerability in unhygienic and unclean environments, and findings suggest that children are vulnerable to mental illnesses. Further investigations into identifying vulnerable groups in informal settlements are needed.

#### 4.1.5. Social Cohesion

Social networks tend to be unstable in informal settlements and are particularly volatile when a portion of households receives upgrading. This can increase feelings of frustration and dissatisfaction, and subsequently result in increased crime and vandalism. This highlights the importance of community participation during informal settlement interventions.

#### 4.1.6. Health Education and Awareness

Other indirect factors that can be addressed to improve health in informal areas is through providing health education regarding the factors that cause ill health in informal settlements. Providing education on how to report broken infrastructure and methods that can be used to clean the neighbourhood are other possible ways to indirectly address health in informal settlements.

## 5. Limitations

Available literature adopts a wide range of terminology to describe informal settlements and investigations into health aspects. Other terms, such as “slums”, “townships”, “settlements” and even “communities” were often used within the literature to refer to settings similar to informal settlements. Whilst different terms were employed in the search criteria of this study, including “slums” and the broader term of “informal”, it is possible that other terms colloquially used to describe informal settlements were missed, which may have resulted in omission of relevant studies.

In addition, the absence of multiple reviewers was a limitation for this literature review. However, the search strategy was jointly developed, and co-authors scrutinised the search results carefully and systematically using the specified inclusion and exclusion criteria, including reviewing the full-text in addition to the abstract.

Due to the complexity of informal settlement contexts and the intrinsic link between poverty and sociocultural factors, it is difficult to attribute causality to a single exposure. This highlights the need for further research utilising a systems approach [89,90,91] to understanding the complex interactions between factors relating to the built environment and health.

## 6. Conclusions

While there is a growing interest in research investigating health in the context of informal settlements, there is a paucity of longitudinal research investigating the impact of informal settlement upgrading or housing improvements in SA on beneficiaries’ health over time. This is particularly concerning for a country that has a proliferating number of informal settlements, especially within a global context that places an emphasis on the need to address poor living environments to improve urban health. In addition, there is a shortage of studies investigating NCDs within the context of informal settlement upgrading and intra-urban housing typologies.

The available literature confirms that informal settlements are poor living environments in which there is a higher burden of disease and a high risk of illness. While the causality between health and factors relating to the informal settlement living environments is complex and not yet fully understood, available studies have suggested that efforts to address crowding, improvements to water and sanitation, and the upgrading of housing typology is likely to be associated with improvements in health and wellbeing. However, through upgrading, it is important for all interrelated dimensions of housing to be considered, namely the house, the home, the neighbourhood and community. For example, we highlight gender-related inequalities that may influence health patterns, and the importance of taking these into account in informal settlement improvement strategies. Lastly, there is a need for more disaggregated interoperable intersectoral data across health and other sectors that influence health, such as housing. These data will be important for informing the development and prioritisation of upgrading strategies for optimising health improvements, and for supporting further research that seeks to measure and evaluate the impact of informal settlement upgrades and interventions on local and wider health trends. Future research will need to include a systems approach to explore and understand the interaction between system components of the city and key health outcomes, particularly in rapidly urbanising low-middle to upper-middle income countries.

## Figures and Tables

**Table 1:** Overview of informal settlement characteristics examples and the associated health conditions categorised by the four World Health Organization (WHO) dimensions of housing [19].

	Characteristic	Sub-Characteristic	Possible Outcome	Associated Health Condition	Sources
House	House size/space				
		Crowding	Exposure to infection	Tuberculosis (TB)	[47]
			Socio-emotional vulnerability	Mental health	[45,48]
			Lack of living space	Injury, burns	[49]
	Structure				
		Noise	Affects sleep and concentration	Mental health; irritability	[48,50]
		Damp/leaks	Mould	Respiratory illness e.g., asthma	[18,48,50]
		Ventilation	Exposure to infection	TB	[48,51]
	Inadequate onsite services	Waste	Pests (pesticides), pathogens	Bites, skin conditions	[48,52,53,54,55]
		Sources of energy	Indoor pollution	Exposure to carcinogens	[56]
			Illegal electricity connections	Injury, burns	[57,58]
Home	Living environment	Crowding	Socio-emotional vulnerability	Mental health	[45,48]
	Socioeconomic status				
		Dissatisfaction/frustration	Mental wellbeing, substance abuse e.g., alcohol	Mental health e.g., depression, stress; violence	[44,59]
Neighbourhood	Environmental	Poor site location	Environmental risk e.g., flooding, exposure to heavy metals	Mental health; exposure to carcinogens	[18,43,48]
		Density of houses	Fires; exposure to infection	Burns, injury, infectious diseases	[18,47,48]
	Services	Access to facilities	Limited access to healthcare	HIV, TB, HIV/TB coinfection	[47]
	Inadequate infrastructure				
		Lack of water	Storage of water	Infectious diseases	[60]
		Lack of sanitation	Use of other means, e.g., bush	Gender violence, crime	[59,61]
		Lack of drainage	Contaminated water	Infectious diseases	[42,62,63]
Community	Social networks	Social stressors	Gender inequality	HIV risk; interpersonal violence	[64,65,66]
			Frustration	Crime, substance abuse	[38,59,67,68]

## Supplementary Materials

The following are available online at https://www.mdpi.com/1660-4601/16/19/3608/s1, Table S1: Search strategy undertaken for initial 2017 search, Table S2: Search strategy undertaken for additional search conducted in 2018.
