# Supplementary material for: A Systematised Review of the Health Impact of Urban Informal Settlements and Implications for Upgrading Interventions in South Africa, a Rapidly Urbanising Middle-Income Country
Source: Int J Environ Res Public Health. 2019 Sep 26;16(19):3608. doi: 10.3390/ijerph16193608 (PMC6801583; doi:10.3390/ijerph16193608)
Supplement: Supplementary file 1 [file ijerph-16-03608-s001.pdf]

# Supplement Material:
# Record of Search Strategy

**Question/Topic:** A systematised literature review was conducted to explore existing evidence and knowledge gaps on the association between informal settlement characteristics and health and the impact of informal settlement upgrading on health, within South Africa, an upper-middle income African country.

**Population/Problem:** Informal settlements
**Intervention/Issue:** Upgrading interventions
**Comparison:** Health status before and after upgrading
**Outcome/Evaluation:** Improved health

**Main concepts/keywords/synonyms/ MeSH terms:** upgrading; informal; health; South Africa; informal settlement; informal settlements; slum; slums; wellbeing

**Databases searched:**
Searches were conducted in two databases to identify recent publications – **Web of Science, PubMed**
Limits: **Language English**
Time frame: **1998 - 2018**

Abstracts of identified documents were read and full text of relevant documents were retrieved for further examination to determine appropriateness for review. In-text searches for the terms "informal settlement", "slum", "health", "infectious", "chronic", "non-communicable disease" were conducted to explore relevance of article.

**Inclusion and exclusion criteria:**

*Inclusion criteria:*
Studies were included if they met any of the following criteria:

- Study is specific to the context of South Africa
- Study has an explicit link to informal settlements, and to at least one of the four dimensions of housing within the context of informal settlements (i.e. the house, the home, the neighbourhood, the community)
- Study has an explicit link to health (physical, mental and social wellbeing, as per the WHO's 1946 definition)

*Exclusion criteria:*
Studies were excluded if they met any of the following criteria:

- Published earlier than 1998, as the South African National Housing Code, which incorporates an informal settlement upgrading plan, was implemented in 1997
- Study only linked to poverty or homelessness; no explicit informal settlement link
- Discussion papers; or studies that are still ongoing
- Methodological concerns and/or methodology that was not well described and/or full text was not available

**Reference lists**: were hand-searched in order to identify studies that were possibly relevant to the following concepts:

- South Africa
- Informal settlement(s) / slums
- Health / wellbeing / any specific health outcomes or diseases

The final studies included in the literature review comprised experiments, retrospective case note reviews, case studies, cross-sectional studies, randomised control trials, qualitative studies, and systematic reviews.
Summary of the database searches are provided in Table S1 and Table S2 below.

**Table S1: Search strategy undertaken for initial 2017 search.**

| Database searched | Search terms | Results | Relevant sources | Relevant source refs | Unique sources used | Unique source refs |
|---|---|---|---|---|---|---|
| **2017 SEARCH**<br>**Limits:** English; 1998 – 2017<br>**Exclusion criteria:** In pre-1998; if not specific to context of South Africa; did not have explicit link to informal settlements' did not have an explicit link to health in context of informal settlements | | | | | | |
| **Pub Med** | **Initial Searches using the specified search:** | | | | | |
| | "UPGRADING" AND "INFORMAL" AND "HEALTH" AND "SOUTH AFRICA" | 0 | 0 | - | - | - |
| | **Broadened searches:** | | | | | |
| | "UPGRADING" AND "INFORMAL" AND "HEALTH" | 8 | 0 | - | - | - |
| | "UPGRADING" AND "INFORMAL SETTLEMENT" AND "HEALTH" | 1 | 0 | - | - | - |
| | "UPGRADING" AND "INFORMAL SETTLEMENTS" AND "HEALTH" | 2 | 0 | - | - | - |
| | "UPGRADING" AND "SLUMS" AND "HEALTH" | 10 | 0 | - | - | - |
| | "UPGRADING" AND "SLUM" AND "HEALTH" | 12 | 0 | - | - | - |
| | "UPGRADING" AND "SLUM" AND "WELLBEING" | 1 | 0 | - | - | - |
| | "UPGRADING" AND "SLUM" AND "WELL BEING" | 3 | 0 | - | - | - |
| | "UPGRADING" AND "INFORMAL" AND "WELLBEING" | 0 | 0 | - | - | - |
| | "UPGRADING" AND "INFORMAL" AND "WELL-BEING" | 2 | 0 | - | - | - |
| | "UPGRADING" AND "INFORMAL" AND "WELL BEING" | 2 | 0 | - | - | - |
| | "UPGRADING" AND "INFORMAL" AND "WELLBEING" | 0 | 0 | - | - | - |
| | **Additional searches using combinations of the following terms** | **57** | **12** | ***[1, 2, 3, 4, 5, 6, 7, 8, 9, 10, 11, 12]*** | **12** | ***[1, 2, 3, 4, 5, 6, 7, 8, 9, 10, 11, 12]*** |
| | "INFORMAL SETTLEMENT", "INFORMAL SETTLEMENTS", "SLUM", "SLUMS", "SOUTH AFRICA", "HEALTH", "WELLBEING", "WELL BEING", "WELL-BEING" | | | | | |
| **Web of Science** | **Initial Search** | | | | | |

| | | | | | | |
|---|---|---|---|---|---|---|
| | “UPGRADING” AND “INFORMAL” AND “HEALTH” AND “SOUTH AFRICA” | 3 | 2 | *[13, 14]* | **2** | *[13, 14]* |
| | **Broadened searches:** | | | | | |
| | “UPGRADING” AND “INFORMAL” AND “HEALTH” | 23 | **3** | *[13, 14, 15]* | **1** | *[15]* |
| | "UPGRADING" AND "INFORMAL SETTLEMENT" AND "HEALTH" | 5 | 2 | *[13, 15]* | - | |
| | "UPGRADING" AND "INFORMAL SETTLEMENTS" AND "HEALTH" | 2 | 0 | **-** | - | - |
| | "UPGRADING" AND "SLUMS" AND "HEALTH" | 17 | 1 | *[15]* | - | - |
| | "UPGRADING" AND "SLUM" AND "HEALTH" | 17 | 0 | - | - | - |
| | "UPGRADING" AND "SLUM" AND "WELLBEING" | 1 | 0 | - | - | - |
| | "UPGRADING" AND "SLUM" AND "WELL BEING" | 5 | 0 | - | - | - |
| | "UPGRADING" AND "INFORMAL" AND "WELLBEING" | 0 | 0 | - | - | - |
| | "UPGRADING" AND "INFORMAL" AND "WELL-BEING" | 2 | 0 | - | - | - |
| | "UPGRADING" AND "INFORMAL" AND "WELL BEING" | 2 | 0 | - | - | - |
| | "UPGRADING" AND "INFORMAL" AND "WELLBEING" | 0 | 0 | - | - | - |
| | **Additional searches using combinations of the following terms:** "INFORMAL SETTLEMENT", “INFORMAL SETTLEMENTS”, “SLUM”, “SLUMS”, "SOUTH AFRICA", "HEALTH”, “WELLBEING”, “WELL BEING”, “WELL-BEING” | **30** | **13** | *[13, 16, 17, 18, 2, 19, 5, 20, 7, 21, 9, 11, 12]* | **6** | *[16, 17, 18, 19, 20, 21]* |
| **REFERENCE LIST** | **Reference lists of selected articles were searched: South Africa; informal settlement(s); slums; any health outcome** | | | | **7** | *[22, 23, 24, 25, 26, 27, 28]* |
| | | | | | **28** | |

## Table S2: Search strategy undertaken for additional search conducted in 2018

# 2018 SEARCH

**Limits:** English; 1998 - 2018

**Excluded Review Round 1:** not specific to South African context (n=83); did not have explicit link to informal settlements (n=157); did not have explicit health link (n=39); discussion papers/studies still ongoing (n=10); methodology not disclosed/full text non available (n=2); duplicated articles (n=23); **Excluded Review Round 2:** if previously included in 2017 search (n=19); not clearly linked to at least 1 of the 4 interrelated dimensions of housing (n=22)

| Database searched | Search terms | Results | Studies retained from round 1 review | Studies retained from round 2 review | Final source refs |
|---|---|---|---|---|---|
| **Pub Med** | ("UPGRADING" AND "INFORMAL" AND "HEALTH" AND "SOUTH AFRICA"); (UPGRADING AND INFORMAL AND HEALTH AND "SOUTH AFRICA"); ("INFORMAL | **152** | **21** | | |

| | | | | | |
|---|---|---|---|---|---|
| | SETTLEMENTS" AND "SOUTH AFRICA" AND HEALTH); ("INFORMAL SETTLEMENT" AND "SOUTH AFRICA" AND “HEALTH”); ("SLUM" AND "SOUTH AFRICA" AND “HEALTH”); (SLUM AND "SOUTH AFRICA" AND “HEALTH”); ("INFORMAL SETTLEMENT" AND "SOUTH AFRICA" AND “WELLBEING”); ("INFORMAL SETTLEMENTS" AND "SOUTH AFRICA" AND “WELLBEING”) | | | | |
| **Web of Science (including MEDLINE and SciELO Citation Index)** | ("UPGRADING" AND "INFORMAL" AND "HEALTH" AND "SOUTH AFRICA"); (UPGRADING AND INFORMAL AND HEALTH AND "SOUTH AFRICA"); ("INFORMAL SETTLEMENTS" AND "SOUTH AFRICA" AND HEALTH); ("INFORMAL SETTLEMENT" AND "SOUTH AFRICA" AND “HEALTH”); ("SLUM" AND "SOUTH AFRICA" AND “HEALTH”); (SLUM AND "SOUTH AFRICA" AND “HEALTH”); ("INFORMAL SETTLEMENT" AND "SOUTH AFRICA" AND “WELLBEING”); ("INFORMAL SETTLEMENTS" AND "SOUTH AFRICA" AND “WELLBEING”) | **221** | **38** | | |
| | ***Round 2 review process*** | | | **18** | ***[29, 30, 31, 32, 33, 34, 35, 36, 37, 38, 39, 40, 41, 42, 43, 44, 45, 46]*** |

## References


1. Gibbs, A.; Washington, L.; Willan, S.; Ntini, N.; Khumalo, T.; Mbatha, N.; Sikweyiya, Y.; Shai, N.; Chirwa, E.; Strauss, M.; et al. The Stepping Stones and Creating Futures Intervention to Prevent Intimate Partner Violence and HIV-Risk Behaviours in Durban, South Africa: Study Protocol for a Cluster Randomized Control Trial, and Baseline Characteristics. BMC Public Health 2017, 17 (1), 336. https://doi.org/10.1186/s12889-017-4223-x.

2. Marais, L.; Sharp, C.; Pappin, M.; Lenka, M.; Cloete, J.; Skinner, D.; Serekoane, J. Housing Conditions and Mental Health of Orphans in South Africa. Heal. Place 2013, 24 (0), 23–29. https://doi.org/10.1016/j.healthplace.2013.08.004.

3. Parbhoo, A.; Louw, Q.; Grimmer-Somers, K. A Profile of Hospital-Admitted Paediatric Burns Patients in South Africa. BMC Res. Notes 2010, 3 (1), 165. https://doi.org/10.1186/1756-0500-3-165.

4. Vearey, J.; Palmary, I.; Thomas, L.; Nunez, L.; Drimie, S. Urban Health in Johannesburg: The Importance of Place in Understanding Intra-Urban Inequalities in a Context of Migration and HIV. Heal. Place 2010, 16 (4), 694–702. https://doi.org/10.1016/j.healthplace.2010.02.007.

5. Scorgie, F.; Vearey, J.; Oliff, M.; Stadler, J.; Venables, E.; Chersich, M. F.; Delany-Moretlwe, S. “Leaving No One behind”: Reflections on the Design of Community-Based HIV Prevention for Migrants in Johannesburg’s Inner-City Hostels and Informal Settlements. BMC Public Health 2017, 17 (1), 482. https://doi.org/10.1186/s12889-017-4351-3.

6. Dalvie, M. A.; Africa, A.; Naidoo, S. Relationship between Firewood Usage and Urinary Cr, Cu and As in Informal Areas of Cape Town. South African Med. J. 2014, 104 (1), 61–64. https://doi.org/10.7196/SAMJ.6451.

7. Tolosana, S.; Rother, H.-A.; London, L. Child’s Play: Exposure to Household Pesticide Use among Children in Rural, Urban and Informal Areas of South Africa. South African Med. J. 2009, 99 (3).

8. Butchart, A.; Kruger, J.; Lekoba, R. Perceptions of Injury Causes and Solutions in a Johannesburg Township: Implications for Prevention. Soc. Sci. Med. 2000, 50 (3), 331–344. https://doi.org/10.1016/S0277-9536(99)00272-5.

9. Gibbs, A.; Govender, K.; Jewkes, R. An Exploratory Analysis of Factors Associated with Depression in a Vulnerable Group of Young People Living in Informal Settlements in South Africa. Glob. Public Health 2016, 0 (0), 1–16. https://doi.org/10.1080/17441692.2016.1214281.

10. Gibbs, A. Tackling Gender Inequalities and Intimate Partner Violence in the Response to HIV: Moving towards Effective Interventions in Southern and Eastern Africa. African J. AIDS Res. 2016, 15 (2), 141–148. https://doi.org/10.2989/16085906.2016.1204331.

11. McLaren, Z. M.; Schnippel, K.; Sharp, A. A Data-Driven Evaluation of the Stop TB Global Partnership Strategy of Targeting Key Populations at Greater Risk for Tuberculosis. PLoS One 2016, 11 (10), 1–12.

https://doi.org/10.1371/journal.pone.0163083.

12. Connolly, C.; Colvin, M.; Shishana, O.; Stoker, D. Epidemiology of HIV in South Africa - Results of a National, Community-Based Survey. South African Med. J. 2004, 94 (9), 776–781.

13. Shortt, N. K.; Hammett, D. Housing and Health in an Informal Settlement Upgrade in Cape Town, South Africa. J. Hous. Built Environ. 2013, 28 (4), 615–627. https://doi.org/10.1007/s10901-013-9347-4.

14. Marais, L.; Ntema, J. The Upgrading of an Informal Settlement in South Africa: Two Decades Onwards. Habitat Int. 2013, 39 (July 2013), 85–95. https://doi.org/10.1016/j.habitatint.2012.11.001.

15. Marais, L.; Cloete, J. "Dying to Get a House?" The Health Outcomes of the South African Low-Income Housing Programme. Habitat Int. 2014, 43, 48–60. https://doi.org/10.1016/j.habitatint.2014.01.015.

16. De Wet, T.; Plagerson, S.; Harpham, T.; Mathee, A. Poor Housing, Good Health: A Comparison of Formal and Informal Housing in Johannesburg, South Africa. Int. J. Public Health 2011, 56 (6), 625–633. https://doi.org/10.1007/s00038-011-0269-1.

17. van Rooyen, J. M.; Kruger, H. S.; Huisman, H. W.; Wissing, M. P.; Margetts, B. M.; Venter, C. S.; Vorster, H. H. An Epidemiological Study of Hypertension and Its Determinants in a Population in Transition: The THUSA Study. J. Hum. Hypertens. 2000, 14 (12), 779–787. https://doi.org/10.1038/sj.jhh.1001098.

18. Marara, T.; Palamuleni, L. G.; Ebenso, E. E. Chemical and Radiological Risks of Drinking Water from Communities in Wonderfonteinspruit Catchment, South Africa. Asian J. Chem. 2013, 25 (16), 9302–9308. https://doi.org/10.14233/ajchem.2013.15502.

19. Narsai, P.; Taylor, M.; Jinabhai, C.; Stevens, F. Variations in Housing Satisfaction and Health Status in Four Lower Socio-Economic Housing Typologies in the EThekwini Municipality in KwaZulu-Natal. Dev. South. Afr. 2013, 30 (3), 367–385. https://doi.org/10.1080/0376835X.2013.817304.

20. Marais, L.; Mehlomakhulu, T. Seriously Ill? Diagnosing the State of Medical Geography in South Africa. South African Geogr. J. 2016, 98 (3), 439–449. https://doi.org/10.1080/03736245.2016.1208584.

21. Abia, A. L. K.; Ubomba-Jaswa, E.; Momba, M. N. B. Prevalence of Pathogenic Microorganisms and Their Correlation with the Abundance of Indicator Organisms in Riverbed Sediments. Int. J. Environ. Sci. Technol. 2016, 13 (12), 2905–2916. https://doi.org/10.1007/s13762-016-1116-y.

22. Westaway, M. S. A Longitudinal Investigation of Satisfaction with Personal and Environmental Quality of Life in an Informal South African Housing Settlement, Doornkop, Soweto. Habitat Int. 2006, 30 (1), 175–189. https://doi.org/10.1016/j.habitatint.2004.09.003.

23. Mathee, A.; Harpham, T.; Barnes, B.; Swart, A.; Naidoo, S.; de Wet, T.; Becker, P. Inequity in Poverty: The Emerging Public Health Challenge in Johannesburg. Dev. South. Afr. 2009, 26 (5), 721–732. https://doi.org/10.1080/03768350903303266.

24. Govender, T.; Barnes, J. M.; Pieper, C. H. Housing Conditions, Sanitation Status and Associated Health Risks in Selected Subsidized Low-Cost Housing Settlements in Cape Town, South Africa. Habitat Int. 2011, 35 (2), 335–342. https://doi.org/10.1016/j.habitatint.2010.11.001.

25. Goebel, A.; Dodson, B.; Hill, T. Urban Advantage or Urban Penalty? A Case Study of Female-Headed Households in a South African City. Health Place 2010, 16 (3), 573–580. https://doi.org/10.1016/j.healthplace.2010.01.002.

26. Bekker, L.; Wood, R. The Changing Natural History of Tuberculosis and HIV Coinfection in an Urban Area of Hyperendemicity. Clin. Infect. Dis. 2010, 50 (s3), S208–S214. https://doi.org/10.1086/651493.

27. Smit, J.; Myer, L.; Middelkoop, K.; Seedat, S.; Wood, R.; Bekker, L.; Stein, D. J.; Town, C.; Africa, S. Mental Health and Sexual Risk Behaviours in a South African Township: A Community-Based Cross-Sectional Study. 2006, 534–542. https://doi.org/10.1016/j.puhe.2006.01.009.

28. Kamndaya, M.; Thomas, L.; Vearey, J.; Sartorius, B.; Kazembe, L. Material Deprivation Affects High Sexual Risk Behavior among Young People in Urban Slums, South Africa. 2014, 91 (3), 581–591. https://doi.org/10.1007/s11524-013-9856-1.

29. Sieber, C.; Ragettli, M. S.; Brink, M.; Olaniyan, T.; Baatjies, R.; Saucy, A.; Vienneau, D.; Probst-Hensch, N.; Dalvie, M. A.; Röösli, M. Comparison of Sensitivity and Annoyance to Road Traffic and Community Noise between a South African and a Swiss Population Sample. Environ. Pollut. 2018, 241, 1056–1062. https://doi.org/10.1016/j.envpol.2018.06.007.

30. Sieber, C.; Ragettli, M. S.; Brink, M.; Toyib, O.; Baatjies, R.; Saucy, A.; Probst-Hensch, N.; Dalvie, M. A.; Roosli, M. Land Use Regression Modeling of Outdoor Noise Exposure in Informal Settlements in Western Cape, South Africa. Int. J. Environ. Res. Public Health 2017, 14 (10). https://doi.org/10.3390/ijerph14101262.

31. Brown-Luthango, M.; Reyes, E.; Gubevu, M. Informal Settlement Upgrading and Safety: Experiences from Cape Town, South Africa. J. Hous. Built Environ. 2017, 32 (3), 471–493. https://doi.org/10.1007/s10901-016-9523-4.

32. Ngole-Jeme, V. M.; Fantke, P. Ecological and Human Health Risks Associated with Abandoned Gold Mine Tailings Contaminated Soil. PLoS One 2017, 12 (2), e0172517. https://doi.org/10.1371/journal.pone.0172517.

33. Collishaw, S.; Gardner, F.; Lawrence Aber, J.; Cluver, L. Predictors of Mental Health Resilience in Children Who Have Been Parentally Bereaved by AIDS in Urban South Africa. J. Abnorm. Child Psychol. 2016, 44 (4), 719–730. https://doi.org/10.1007/s10802-015-0068-x.

34. Van Niekerk, A.; Govender, R.; Hornsby, N.; Swart, L. Household and Caregiver Characteristics and Behaviours as Predictors of Unsafe Exposure of Children to Paraffin Appliances. Burns 2017, 43 (4), 866–876.

https://doi.org/10.1016/j.burns.2016.10.022.

35. Odendaal, W.; van Niekerk, A.; Jordaan, E.; Seedat, M. The Impact of a Home Visitation Programme on Household Hazards Associated with Unintentional Childhood Injuries: A Randomised Controlled Trial. Accid. Anal. Prev. 2009, 41 (1), 183–190. https://doi.org/10.1016/j.aap.2008.10.009.

36. Archer, C. E.; Schoeman, M. C.; Appleton, C. C.; Mukaratirwa, S.; Hope, K. J.; Matthews, G. B. Predictors of Trypanosoma Lewisi in Rattus Norvegicus from Durban, South Africa. J. Parasitol. 2018, 104 (3), 187–195. https://doi.org/10.1645/17-92.

37. Balme, K. H.; Roberts, J. C.; Glasstone, M.; Curling, L.; Rother, H.-A.; London, L.; Zar, H.; Mann, M. D. Pesticide Poisonings at a Tertiary Children's Hospital in South Africa: An Increasing Problem. Clin. Toxicol. (Phila). 2010, 48 (9), 928–934. https://doi.org/10.3109/15563650.2010.534482.

38. De Klerk, P.; Dijk, M. Van; As, A. B. Van. Treatment and Outcome of Unusual Animal Bite Injuries in Young Children. South African Med. J. 2016, 106 (2), 206–209. https://doi.org/10.7196/SAMJ.2016.v106i2.10106.

39. Abia, A. L. K.; Alisoltani, A.; Keshri, J.; Ubomba-Jaswa, E. Metagenomic Analysis of the Bacterial Communities and Their Functional Profiles in Water and Sediments of the Apies River, South Africa, as a Function of Land Use. Sci. Total Environ. 2018, 616–617, 326–334. https://doi.org/10.1016/j.scitotenv.2017.10.322.

40. Abia, A. L. K.; Ubomba-Jaswa, E.; du Preez, M.; Momba, M. N. B. Riverbed Sediments in the Apies River, South Africa: Recommending the Use of Both Clostridium Perfringens and Escherichia Coli as Indicators of Faecal Pollution. J. Soils Sediments 2015, 15 (12), 2412–2424. https://doi.org/10.1007/s11368-015-1209-0.

41. Olaniran, A. O.; Naicker, K.; Pillay, B. Antibiotic Resistance Profiles of Escherichia Coli Isolates from River Sources in Durban, South Africa. World J. Microbiol. Biotechnol. 2009, 25 (10), 1743–1749. https://doi.org/10.1007/s11274-009-0071-x.

42. Paulse, A. N.; Jackson, V. A.; Khan, W. Comparison of Microbial Contamination at Various Sites along the Plankenburg-and Diep Rivers, Western Cape, South Africa. Water SA 2009, 35 (4), 469–478.

43. Gibbs, A.; Dunkle, K.; Washington, L.; Willan, S.; Shai, N.; Jewkes, R. Childhood Traumas as a Risk Factor for HIV-Risk Behaviours amongst Young Women and Men Living in Urban Informal Settlements in South Africa: A Cross-Sectional Study. PLoS One 2018, 13 (4), e0195369. https://doi.org/10.1371/journal.pone.0195369.

44. Gibbs, A.; Jewkes, R.; Willan, S.; Washington, L. Associations between Poverty, Mental Health and Substance Use, Gender Power, and Intimate Partner Violence amongst Young (18-30) Women and Men in Urban Informal Settlements in South Africa: A Cross-Sectional Study and Structural Equation Model. PLoS One 2018, 13 (10), 1–19. https://doi.org/10.1371/journal.pone.0204956.

45. Gibbs, A.; Dunkle, K.; Jewkes, R. Emotional and Economic Intimate Partner Violence as Key Drivers of Depression and Suicidal Ideation: A Cross-Sectional Study among Young Women in Informal Settlements in South Africa. PLoS One 2018, 13 (4), e0194885. https://doi.org/10.1371/journal.pone.0194885.

46. Gibbs, A.; Sikweyiya, Y.; Jewkes, R. "Men Value Their Dignity": Securing Respect and Identity Construction in Urban Informal Settlements in South Africa. Glob. Health Action 2014, 7, 23676.
